# Insulin-like growth factor 1 and risk of depression in older people: the English Longitudinal Study of Ageing

**DOI:** 10.1038/tp.2016.167

**Published:** 2016-09-20

**Authors:** S Chigogora, P Zaninotto, M Kivimaki, A Steptoe, G D Batty

**Affiliations:** 1 Department of Epidemiology and Public Health, University College London, London, UK; 2 Centre for Cognitive Ageing and Cognitive Epidemiology, University of Edinburgh, Edinburgh, UK

## Abstract

Depressive disorders are a leading cause of disability in older age. Although the role of psychosocial and behavioural predictors has been well examined, little is known about the biological origins of depression. Findings from animal studies have implicated insulin-like growth factor 1 (IGF-1) in the aetiology of this disorder. A total of 6017 older adults (mean age of 65.7 years; 55% women) from the English Longitudinal Study of Ageing provided serum levels of IGF-1 (mean=15.9 nmol l^−1^, s.d. 5.7) during a nurse visit in 2008. Depression symptoms were assessed in the same year and again in 2012 using the eight-item Center for Epidemiologic Studies Depression Scale. Self-reports of a physician-diagnosis of depression were also collected at both time points. In separate analyses for men and women, the results from both the cross-sectional and longitudinal analyses revealed a ‘U'-shaped pattern of association, such that lower and higher levels of IGF-1 were associated with a slightly elevated risk of depression, whereas the lowest risk was seen around the median levels. Thus, in men, with the lowest quintile of IGF-1 as the referent, the age-adjusted odds ratios (95% confidence interval) of developing depression symptoms after 4 years of follow-up, for increasing quintiles of IGF-1, were: 0.51 (0.28–0.91), 0.50 (0.27–0.92), 0.63 (0.35–1.15) and 0.63 (0.35–1.13) (*P*-value for quadratic association 0.002). Some attenuation of these effects was apparent after adjustment for co-morbidity, socioeconomic status and health behaviours. In conclusion, in the present study of older adults, there was some evidence that moderate levels of IGF-1 levels conferred a reduced risk of depression.

## Introduction

Depression, a major public health problem, has profound effects on the economy and on individuals. According to the World Health Organization, it is the leading cause of disability worldwide,^1^ responsible for 65 million disability-adjusted life years in 2008.^2^ In addition to being an important disease in its own right, depression is also associated with an increased risk of suicide,^3, 4^ cardiovascular disease,^5, 6, 7^ some cancers^8^ and premature mortality.^9, 10, 11^ Although effective treatments for depression are available, the majority of cases among older people remain undiagnosed; prevention of the disorder is therefore crucial.^12, 13^ A series of studies have identified a range of behavioural (heavy alcohol use, some dietary characteristics) and social (loneliness, socioeconomic disadvantage, bereavement) predictors of depression in later life.^14, 15^ Although the aetiological role of weight, height, genetic factors and several plasma markers has been examined,^16, 17, 18^ relatively less attention has been paid to biological risk factors.

Insulin-like growth factor 1 (IGF-1) is a complex peptide hormone produced in multiple tissue sites whose main function is to mediate cell growth, differentiation and transformation by promoting mitosis and inhibiting apoptosis.^19^ Evidence from laboratory studies suggests that expression and function of IGF-1 is similar in humans and rodents.^20, 21^ In humans, elevated IGF-1 levels are associated with increased skeletal muscle and tissue growth,^22, 23^ bone mineral density,^24^ risk of selected malignancies^25^ and cardiovascular disease.^26, 27^ IGF-1 has also been implicated in psychological functioning of both animals and humans. Thus, rodents whose circulating IGF-1 levels were experimentally perturbed displayed marked changes in mood. For instance, the application of a viral vector to drastically reduce circulating IGF-1 levels resulted in mice showing signs of depression as assessed using the forced swim and tail suspension tests.^28^ Elsewhere, rodents given IGF-1 infusions experienced a reduction in depression symptoms relative to a no treatment group.^29, 30^ In laboratory-based experimental studies of humans, through a range of mechanisms such as altered neuroprotection, modulation of neuronal excitability, brain angiogenesis, hippocampal neurogenesis and neuroplasticity,^31, 32, 33^ alterations of IGF-1 levels have been linked to hippocampal dysfunction, which in turn have been associated with mood disorders.^34, 35^ IGF-1 expression in the hippocampus has also been found to be reduced in sufferers of depression, and enhanced by the administration of antidepressants.^36^

Of the two general population-based studies of which we are aware, one showed that low IGF-1 in women, but high levels in men, were predictive of depressive disorder.^37^ In another, relative to moderate levels, women with high IGF-1 values experienced an elevated risk of minor depression.^38^ In both the studies, incident depression was rare resulting in low statistical power, and in one,^38^ the measurement of depression was made using a non-standard scale of unknown validity. Accordingly, against this background of study paucity, discordant findings and methodological concerns, we examined the cross-sectional and longitudinal association between IGF-1 and depression symptoms in a large, well-established general population-based study of older adults in England (UK).

## Materials and methods

### Study population

The English Longitudinal Study of Ageing is an ongoing prospective cohort study of adults aged 50 years and over who, when recruited, lived in private households in the United Kingdom. Initiated in 2002/3, the original sample was drawn from participants in the Health Surveys for England, a collection of population-based cross-sectional studies. With data collection occurring every 2 years, as of 2012, there have been a total of six waves. As IGF-1 levels were first measured in 2008 (wave 4), this represents our study ‘baseline' for the purposes of the present analyses. Ethical approval for all data collection was granted by the National Research and Ethics Committee,^39^ and the participants provided written consent.

### Measurement of IGF-1

Study members were requested not to eat, smoke, drink alcohol or engage in vigorous exercise for 30 min before blood being drawn. The whole-blood samples were transported to a single laboratory (Royal Victoria Infirmary, Newcastle, UK), where the serum was separated, frozen at 40 °C and batch-assayed (completed in 2008) using the DPC Immulite 2000 method. The inter-assay coefficient of variation for IGF-1 across a range of levels was ⩽3.7%, and the intra-assay coefficient of variation was ⩽5.3%. The IGF-1 values are reported as whole numbers (range: 3–200 nmol l^−1^).^40^

### Depressive symptoms and physician-diagnosis of depression

Depression symptoms were ascertained during a computer-assisted personal interview using the eight-item Center for Epidemiologic Studies Depressive (CES-D8) scale.^41^ Each item requires a dichotomous (yes/no) response, and scores range between 0 and 8 (higher score denotes more severe symptoms). Consistent with other analyses, we defined ‘caseness' as anyone scoring 4 or above.^42^ The CES-D has been widely used in population-based studies of older groups^43, 44^ and has been validated against clinician-assessed depression.^45^ Notably, the shortened CES-D8 has good internal consistency (Cronbach's *α*=0.78) and similar psychometric properties to the full 20-item CES-D.^46^ The participants also had an opportunity to self-report a physician-diagnosis of depression. During the main English Longitudinal Study of Ageing interview, the participants first reported whether they had ever been diagnosed with any emotional, nervous or psychiatric problems. This was followed by the identification of the actual condition as selected from a list of ailments common to this group, of which depression was one.^47^ The assessment of depression in this manner has been shown to be valid in a separate study using the Structured Clinical Interview for DSM-IV Axis I Disorders as the gold standard.^48^ Both these measurements of depression were made in 2008 and 2012.

### Measurement of covariates

We grouped covariates, including potential confounders and mediators, according to theme. Anthropometric measures comprised height and weight, which were measured during the nurse visit; body mass index (kg/m^2^) calculated by dividing each individual's measured weight by height squared. Psychosocial factors were level of education (no qualification, completed secondary (high school) education, educated beyond secondary education but below degree level, and educated beyond degree level); quintiles of net non-pension wealth (derived from an estimation of financial wealth and physical assets reported by study participants and their partners, excluding pension savings and net of debts such as credit cards and loans);^49^ and marital status (currently living with a partner or not). Health-related behaviours comprised smoking status (current, ex-smoker and never), frequency of alcohol consumption in the past year (less than daily/daily, with consumption on at least 5 days of the week being classed as daily consumption) and leisure time physical activity (low/sedentary, moderate or high activity of exercises such as jogging, cycling, gardening, walking). Co-morbidities were self-reported physician-diagnosis of cancer, diabetes or cardiovascular disease (heart murmur, ischaemic heart disease, abnormal heart rhythm, stroke, valvular heart disease or any other reported heart disease).

### Statistical analysis

**Figure 1:**
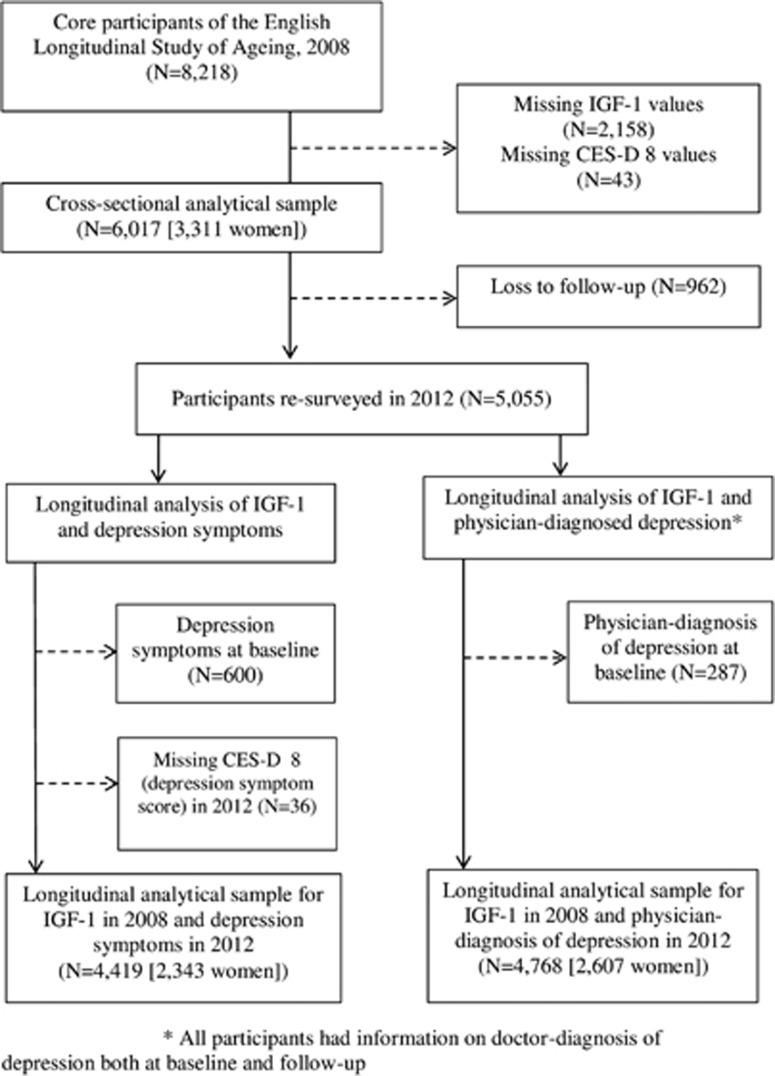
Derivation of the analytical sample for cross-sectional (2008) and longitudinal (2012) analyses of the association between serum IGF-1 and depression: the English Longitudinal Study of Ageing. CES-D, Center for Epidemiologic Studies Depressive scale; IGF-1, insulin-like growth factor 1.

In Figure 1, we illustrate the flow of participants through the study. Of the 8218 participants at baseline in 2008 who had received a nurse visit, we excluded those who had missing values for IGF-1 (*N*=2158) comprising people who declined to give blood (771), unsuitability or loss of a blood sample (395), or failure to obtain blood sample (992). We also excluded participants with missing values for depression symptoms (43), although none had missing data for physician-diagnosed depression at baseline. The cross-sectional sample therefore comprised 6017 study members from data collection in 2008. We also carried out the longitudinal analyses, again using IGF-1 values from 2008 but relating to new (incident) cases of depression at resurvey in 2012. In deriving new cases of depression, we excluded participants who were classed as depression cases in 2008, resulting in samples of 4419 for the analysis of depression symptoms and 4768 for physician-diagnosed depression.

Multivariable logistic regression analyses were used to summarize the association of IGF-1 levels with both depression outcomes. The lowest quintile of IGF-1 was used as the referent. There is existing evidence of differential IGF-1-depression relationships in men and women,^39, 40, 50^ so we present gender-specific analyses here also. We adjusted effect estimates for known covariates in a stepwise manner. In our analyses, depression symptoms were our primary outcome, with physician-diagnosed depression used to test convergence of evidence. All the analyses were carried out using Stata12SE software.^51^

## Results

### IGF-1 and baseline characteristics: cross-sectional analyses

In Table 1 (women) and Table 2 (men), we present baseline study participant characteristics according to IGF-1 quintiles. As expected, mean IGF-1 values were higher in men (16.5 nmol l^−1^) than women (15.2 nmol l^−1^). In men and women, IGF-1 was inversely associated with age and directly related to height and socioeconomic position. Psychosocial factors, which included social position and cohabiting with a partner, typically occurred at more favourable levels in men and women with higher IGF-1 values. A total of 776 participants (12.9%) had CES-D8 scores of 4 and above at baseline and were therefore denoted as ‘cases' (71.8% were female); 344 (5.7%) participants reported physician-diagnosis of depression. As for some of the somatic conditions such as cancer, there was a suggestion that the greatest proportions of both men and women who reported high depression symptoms and self-declared physician-diagnosed depression were seen in the lowest and highest quintiles of IGF-1, although the differences across groups were not considerable.

### IGF-1 and depression: cross-sectional analyses

In Table 3, the computation of odds ratios for the cross-sectional association between IGF-1 and depression symptoms supports earlier evidence of a somewhat higher risk of depression symptoms at opposite ends of the IGF-1 continuum in men and women in this study. Although a similar 'U'-shaped pattern was apparent for physician-diagnosed depression (see Supplementary Table 1), statistical significance at conventional levels was rarely apparent for individual point estimates in these analyses. Adjustment for an array of covariates had little impact on this pattern of association, although taking into account all covariates simultaneously led to some flattening of the IGF-1–depression relationship.

### IGF-1 and depression: prospective analyses

In Table 4, we depict the association between IGF-1 and depression symptoms after 4 years of follow-up in participants initially free of depression symptoms at baseline (longitudinal analyses). A ‘U'-shaped pattern of risk was again observed for the IGF-1–depression association in both genders based on symptomatology. Similar results were apparent for physician-diagnosis of depression but only among women (Supplementary Table 2). Statistical significance was, again, rarely apparent for individual point estimates. In none of our analyses was there strong statistical evidence that gender modified the IGF-1–depression association (*P*-values for interaction for the multiply adjusted odds of developing depression symptoms and physician-diagnosed depression in longitudinal analyses are 0.531 and 0.275, respectively).

## Discussion

The main finding of this study of older people was that having IGF-1 levels at opposing ends of the continuum was associated with a slightly higher risk of depression symptoms. Similar results were apparent for physician-diagnosis of depression.

### Comparison with existing studies

Our results partially accord with the two population studies on IGF-1 and depression of which we are aware. When compared with data from the Study of Health in Pomerania in Germany,^38^ our results were in agreement with the finding that having a low IGF-1 level was associated with higher risk of developing depression symptoms among women. However, we also found a similar association for men. Our results are also in agreement with the second existent study, which used data from The Longitudinal Aging Study Amsterdam,^39^ where associations were found between median levels of IGF-1 and lower risk of both prevalent and incident depression symptoms. Once again, the main difference with this present study is that we identified this association among both men and women. The ‘U'-shaped relationship that we identified between IGF-1 and depression symptoms is supported by observations of increased reports of lifetime affective disorders in individuals with lower (pituitary dwarfism) and higher (acromegaly) levels of this growth hormone.^52, 53, 54^ It may be that this apparent differential result for men and women in these existing studies is due to statistical instability.

To directly compare our findings with some of those already published, we re-categorized IGF-1 levels in our own analyses. In these new analyses, we still found evidence for increased odds of depression symptoms among those with very low and very high IGF-1. Our results after initial re-categorization of IGF-1 levels^38^ showed that, after 4 years of follow-up, the age-adjusted odds ratios (95% confidence interval) of depression symptoms for the lowest and highest tenth percentiles of IGF-1 among men were 2.26 (1.25, 4.09) and 1.51 (0.86, 2.68), respectively when compared with those with intermediate levels; whereas the corresponding results in women were 1.38 (0.93, 2.07) and 1.27 (0.77, 2.10). Following further re-categorization,^39^ when compared with the middle tertile, the odds ratios (95% confidence interval) of depression for the lowest and highest tertiles of IGF-1 among men were 1.14 (0.72, 1.80) and 0.89 (0.55, 1.43), respectively, and corresponding results for women were 1.08 (0.79, 1.49) and 1.14 (0.79, 1.65).

Our findings, however, contrast with the results from the handful of case–control studies where IGF-1 levels were found to be elevated in depressed patients compared with healthy controls.^55, 56^ This may be owing to specific consequences of previous use of anti-depressant medications, such as where they have been seen to improve expression of IGF-1 and other neutrophic and growth factors in the hippocampus.^31^ Furthermore, there may yet be other unknown biological mechanisms related to the state of being depressed, which cause serum IGF-1 levels to increase, implying reverse causation in the reported case–control studies, where the depression in the cases had, in fact, caused the IGF-1 levels to increase. In this study, we attempted to circumvent the problem of reverse causality by utilizing depression incidence as our outcome in the longitudinal analyses; that is, new cases of depression in participants who, at baseline, were symptom-free and had not previously reported being diagnosed with the condition by a physician.

### Strengths and limitations

The main strength of this study is that it has a large, nationally representative sample of people aged 50 years plus in whom there were high rates of follow-up when two standard measures of depression were administered. Our study is not of course without its limitations. The observational nature of our study indicates that we are not able to make any assertions about cause and effect. Although suggestions for mechanisms of action have been posited, it remains possible that IGF-1 levels are a proxy for other factors that are causally related to depression (residual confounding). Although our study was very well characterized, we are not able to control for all possible confounders. Furthermore, although the CES-D8 is a widely used questionnaire in observational studies, it does not provide a diagnosis of depression. Conversely, although self-reported physician-diagnosis of depression does, in principle, do this, many people with depression do not seek medical intervention. The use of anti-depressant medication, which also has some utility in identifying study members with a depression diagnosis, was not gathered in English Longitudinal Study of Ageing. It is also the case, however, that administration of such therapy does not necessarily imply a diagnosis of depression: anti-depressant medication can be used in the treatment of, among other conditions, anxiety and chronic pain disorders. The occurrence of missing data is inevitable in any large-scale study, and about 10% of participants had missing data for one or more of the covariates. However, sensitivity analysis comparing results across the cases with complete information and those with some missing covariates made little difference to outcomes, suggesting that major bias is unlikely. Finally, severe liver and kidney disease may influence IGF-I levels, but we had no such data on these morbidities herein.

In conclusion, taken together, in the present study of older adults, having IGF-1 values at opposite ends of the continuum was associated with a somewhat increased risk of depression symptoms and physician-diagnosis of depression. Further studies are needed to examine whether the observed association is likely to be causal before meaningful discussions about normalizing IGF-1 levels with drugs could be useful in the prevention of depressive symptoms in older people.

## Figures and Tables

**Table 1:** Baseline characteristics of study participants according to quintiles of serum IGF-1 (nmol l^−1^): 3311 women in the English Longitudinal Study of Ageing, 2008

	*IGF-1 quintile (range)*						P*-value for difference*
	*1*	*2*	*3*	*4*	*5*	*All*	
	*(2–11 nmol l^−1^)*	*(12–14 nmol l^−1^)*	*(15–16 nmol l^−1^)*	*(17–20 nmol l^−1^)*	*(21–65 nmol l^−1^)*		
Subject numbers	861	813	479	681	477		
IGF-1, nmol l^−1^, mean (s.e.)	9.2 (0.06)	13.0 (0.03)	15.5 (0.02)	18.3 (0.04)	25.2 (0.22)	15.2 (0.10)	<0.001
Age, years, mean (s.e.)	68.7 (0.4)	66.2 (0.3)	65.1 (0.4)	64.4 (0.3)	63.2 (0.4)	65.9 (0.2)	<0.001
							
*Anthropometry mean (s.e.)*							
Height, cm	158.8 (0.3)	159.9 (0.2)	160.4 (0.3)	160.6 (0.2)	160.9 (0.3)	160.0 (0.1)	<0.001
BMI, kg/m^2^	28.7 (0.2)	28.4 (0.2)	27.6 (0.2)	27.7 (0.2)	27.6 (0.2)	28.1 (0.1)	<0.001
							
*Co-morbidities, % (s.e.)*							
Diabetes	8.5 (0.9)	6.0 (0.8)	6.9 (1.2)	5.9 (0.9)	7.8 (1.2)	7.0 (0.4)	0.213
Cancer	6.6 (0.8)	4.5 (0.7)	4.6 (1.0)	3.2 (0.7)	6.1 (1.1)	5.0 (0.4)	0.029
Cardiovascular disease	26.6 (1.5)	24.4 (1.5)	21.5 (1.9)	18.6 (1.5)	23.9 (2.0)	23.3 (0.7)	0.005
							
*Psychosocial factors, % (s.e.)*							
Lowest wealth quintile	23.1 (1.4)	16.0 (1.3)	11.7 (1.5)	15.0 (1.4)	14.5 (1.6)	16.8 (0.7)	<0.001
No educational qualifications	34.1 (1.6)	30.3 (1.6)	29.0 (2.1)	27.2 (1.7)	23.1 (1.9)	29.4 (0.8)	0.001
Lives alone	43.1 (1.7)	37.5 (1.7)	28.4 (2.1)	32.8 (1.8)	36.3 (2.2)	36.5 (0.8)	<0.001
							
*Behavioural factors, % (s.e.)*							
Sedentary or low physical activity	39.4 (1.7)	30.0 (1.6)	26.5 (2.0)	25.8 (1.7)	30.2 (2.1)	31.1 (0.8)	<0.001
Current smoking	12.2 (1.1)	13.3 (1.2)	12.7 (1.5)	13.5 (1.3)	16.1 (1.7)	13.4 (0.6)	0.663
Daily alcohol intake	15.2 (1.2)	16.0 (1.3)	16.5 (1.7)	15.9 (1.4)	16.8 (1.7)	16.0 (0.6)	0.902
							
*Depression*							
CES-D score, mean (s.e.)	1.8 (0.07)	1.6 (0.07)	1.3 (0.08)	1.5 (0.07)	1.6 (0.09)	1.6 (0.03)	<0.001
High depression symptoms, % (s.e.)	20.6 (1.3)	15.9 (1.3)	14.4 (1.6)	14.2 (1.3)	17.8 (1.5)	16.8 (0.7)	0.005
Physician-diagnosed depression, % (s.e.)	7.1 (0.9)	6.4 (0.9)	5.4 (1.0)	6.5 (0.9)	6.7 (1.1)	6.5 (0.4)	0.837

Abbreviations: BMI, body mass index; CES-D, Center for Epidemiologic Studies Depressive scale; IGF-1, insulin-like growth factor 1.

**Table 2:** Baseline characteristics of study participants according to quintiles of serum IGF-1 (nmol l^−1^): 2706 men in the English Longitudinal Study of Ageing, 2008

	*IGF-1 quintile (range)*						P*-value for difference*
	*1*	*2*	*3*	*4*	*5*	*All*	
	*(2–11 nmol l^−1^)*	*(12–14 nmol l^−1^)*	*(15–16 nmol l^−1^)*	*(17–20 nmol l^−1^)*	*(21–65 nmol l^−1^)*		
Subject number	483	615	384	667	557		
IGF-I, nmol l^−1^ (s.e.)	9.3 (0.08)	13.1 (0.03)	15.5 (0.03)	18.4 (0.04)	25.1 (0.22)	16.5 (0.11)	<0.001
Age, years, mean (s.e.)	68.7 (0.5)	65.3 (0.4)	65.0 (0.5)	64.4 (0.3)	64.0 (0.3)	65.4 (0.2)	<0.001
							
*Anthropometry, mean (s.e.)*							
Height, cm	171.7 (0.3)	172.8(0.3)	173.8 (0.4)	173.8 (0.3)	174.2 (0.3)	173.3 (0.1)	<0.001
BMI, kg/m^2^	28.6 (0.2)	27.8 (0.2)	27.9 (0.2)	28.0 (0.2)	27.9 (0.2)	28.0 (0.1)	0.078
							
*Co-morbidities, % (s.e.)*							
Diabetes	12.6 (1.5)	6.8 (1.0)	6.3 (1.2)	10.6 (1.2)	11.1 (1.3)	9.6 (5.7)	0.001
Cancer	4.8 (1.0)	5.4 (1.0)	4.4 (1.1)	5.2 (0.9)	4.3 (0.9)	4.9 (4.1)	0.897
Cardiovascular disease	31.5 (2.1)	24.6 (1.7)	25.7 (2.2)	26.3 (1.7)	27.2 (1.9)	26.9 (0.9)	0.121
							
*Psychosocial factors, % (s.e.)*							
Lowest wealth quintile	18.0 (1.8)	13.3 (1.4)	14.3 (1.8)	12.0 (1.3)	13.1 (1.4)	13.9 (0.7)	0.167
No educational qualifications	25.9 (2.0)	20.0 (1.6)	23.4 (2.1)	17.3 (1.5)	16.3 (1.6)	20.1 (0.8)	0.056
Lives alone	26.7 (2.0)	20.7 (1.6)	20.8 (2.1)	21.4 (1.6)	16.7 (1.6)	21.1 (0.8)	0.003
							
*Behavioural factors, % (s.e.)*							
Sedentary or low physical activity	26.9 (2.0)	18.4 (1.6)	22.1 (2.1)	17.4 (1.5)	21.9 (1.8)	20.9 (0.8)	0.005
Current smoking	13.7 (1.6)	12.7 (1.3)	12.8 (1.7)	11.8 (1.3)	14.0 (1.5)	12.9 (0.6)	0.306
Daily alcohol intake	28.6 (2.2)	24.7 (1.7)	27.6 (2.2)	26.4 (1.7)	24.1 (1.8)	26.1 (0.8)	<0.001
							
*Depression*							
CES-D8 score, mean (s.e.)	1.0 (0.08)	0.9 (0.06)	0.8 (0.07)	0.9 (0.06)	1.1 (0.07)	0.9 (0.03)	0.07
High depression symptoms, % (s.e.)	9.3 (1.3)	7.5 (1.1)	5.7 (1.0)	6.7 (1.0)	11.0 (1.3)	8.1 (0.5)	0.018
Physician-diagnosed depression, % (s.e.)	5.2 (1.0)	4.2 (0.8)	4.2 (1.0)	3.9 (0.7)	6.5 (1.0)	4.8 (0.4)	0.238

Abbreviations: BMI, body mass index; CES-D, Center for Epidemiologic Studies Depressive scale; IGF-1, insulin-like growth factor 1.

**Table 3:** Odds ratio (95% confidence interval) for the cross-sectional association between serum IGF-1 and depression symptoms: the English Longitudinal Study of Ageing, 2008

	*IGF-1 quintile (range)*^a^					P*-value for linearity*	P*-value for quadratic*
	*1*	*2*	*3*	*4*	*5*		
	*(2–11)*	*(12–14)*	*(15–16)*	*(17–20)*	*(21–65)*		
*Women (analytical sample)*							
Adjustments							
Age (3311)	1 (ref)	0.76 (0.58, 0.97)	0.69 (0.50, 0.93)	0.68 (0.52, 0.90)	0.91 (0.68, 1.22)	0.027	0.009
Age, anthropometric measures^b^ (3181)	1	0.79 (0.61, 1.02)	0.74 (0.54, 1.01)	0.72 (0.54, 0.95)	0.96 (0.71, 1.30)	0.061	0.037
Age, co-morbidities^c^ (3308)	1	0.75 (0.58, 0.96)	0.68 (0.50, 0.92)	0.69 (0.52, 0.91)	0.89 (0.66, 1.19)	0.031	0.012
Age, psychosocial factors^d^ (3162)	1	0.81 (0.62, 1.06)	0.79 (0.57, 1.09)	0.73 (0.55, 0.98)	0.96 (0.70, 1.30)	0.494	0.026
Age, behavioural factors^e^ (2920)	1	0.77 (0.58, 1.02)	0.76 (0.55, 1.06)	0.76 (0.56, 1.02)	0.91 (0.66, 1.25)	0.08	0.078
Multiply adjusted (2691)	1	0.86 (0.63, 1.16)	0.9 (0.63, 1.29)	0.85 (0.62, 1.29)	1 (0.71, 1.41)	0.756	0.199
							
*Men (analytical sample)*							
Adjustments							
Age (2706)	1 (ref)	0.75 (0.49, 1.16)	0.56 (0.33, 0.95)	0.66 (0.43, 1.02)	1.12 (0.74, 1.69)	0.008	0.004
Age, anthropometric measures^b^ (2627)	1	0.85 (0.54, 1.34)	0.62 (0.35, 1.09)	0.79 (0.50, 1.25)	1.26 (0.81, 1.96)	0.037	0.011
Age, co-morbidities^c^ (2696)	1	0.76 (0.49, 1.18)	0.58 (0.34, 0.98)	0.64 (0.41, 0.99)	1.11 (0.73, 1.68)	0.009	0.003
Age, psychosocial factors^d^ (2592)	1	0.85 (0.54, 1.34)	0.61 (0.35, 1.06)	0.75 (0.47, 1.19)	1.3 (0.90, 2.16)	0.015	0.045
Age, behavioural factors^e^ (2377)	1	0.68 (0.40, 1.16)	0.57 (0.31, 1.06)	0.71 (0.43, 1.18)	1.32 (0.83, 2.12)	<0.001	0.052
Multiply adjusted (2277)	1	0.84 (0.47, 1.50)	0.63 (0.32, 1.24)	0.84 (0.48, 1.47)	1.54 (0.90, 2.64)	0.006	0.153

Abbreviations: BMI, body mass index; IGF-1, insulin-like growth factor 1.

a IGF-1 units are nmol l^−1^.

b BMI, height.

c Cancer, diabetes, cardiovascular disease.

d Own wealth quintile per benefit unit (unit is a couple or single person along with their dependent children), education level.

e Alcohol consumption, smoking, physical activity.

**Table 4:** Odds ratio (95% confidence interval) for the longitudinal association between serum IGF-1 in 2008 and new depression symptoms in 2012: the English Longitudinal Study of Ageing

	*IGF-1 quintile (range)*^a^					P*-value for linearity*	P*-value for quadratic*
	*1*	*2*	*3*	*4*	*5*		
	*(2–11)*	*(12–14)*	*(15–16)*	*(17–20)*	*(21–65)*		
*Women (analytical sample)*							
Adjustments							
Age (2343)	1 (ref)	0.88 (0.60, 1.29)	0.77 (0.51, 1.16)	0.84 (0.53, 1.35)	0.95 (0.60, 1.50)	0.647	0.027
Age, anthropometric measures^b^ (2276)	1	0.84 (0.57, 1.25)	0.76 (0.50, 1.16)	0.84 (0.52, 1.36)	0.93 (0.58, 1.48)	0.652	0.033
Age, co-morbidities^c^ (2342)	1	0.86 (0.58, 1.26)	0.77 (0.51, 1.16)	0.84 (0.53, 1.35)	0.94 (0.60, 1.49)	0.649	0.014
Age, psychosocial factors^d^ (2243)	1	0.9 (0.60, 1.34)	0.86 (0.56, 1.30)	0.9 (0.55, 1.45)	1.06 (0.66, 1.69)	0.816	0.026
Age, behavioural factors^e^ (2116)	1	0.91 (0.61, 1.35)	0.76 (0.49, 1.17)	0.86 (0.52, 1.40)	0.93 (0.58, 1.51)	0.915	0.029
Multiply adjusted (1969)	1	0.86 (0.56, 1.31)	0.78 (0.50, 1.23)	0.87 (0.52, 1.46)	0.94 (0.56, 1.57)	0.922	0.008
							
*Men (analytical sample)*							
Adjustments							
Age (2076)	1 (ref)	0.51 (0.28, 0.91)	0.5 (0.27, 0.92)	0.63 (0.35, 1.15)	0.63 (0.35, 1.13)	0.126	0.002
Age, anthropometric measures^b^ (2042)	1	0.51 (0.28, 0.94)	0.5 (0.27, 0.93)	0.66 (0.36, 1.20)	0.63 (0.34, 1.14)	0.142	0.002
Age, co-morbidities^c^ (2070)	1	0.52 (0.29, 0.94)	0.56 (0.24, 0.85)	0.64 (0.35, 1.16)	0.65 (0.36, 1.16)	0.098	0.002
Age, psychosocial factors^d^ (2031)	1	0.53 (0.29, 0.97)	0.54 (0.29, 0.99)	0.62 (0.33, 1.17)	0.68 (0.37, 1.24)	0.173	0.001
Age, behavioural factors^e^ (2076)	1	0.52 (0.27, 0.99)	0.49 (0.25, 0.96)	0.67 (0.35, 1.30)	0.58 (0.30, 1.11)	0.165	0.001
Multiply adjusted (1765)	1	0.51 (0.25, 1.02)	0.5 (0.22, 0.95)	0.67 (0.33, 1.33)	0.6 (0.30, 1.19)	0.072	0.003

Abbreviations: BMI, body mass index; IGF-1, insulin-like growth factor 1.

a IGF-1 units are nmol l^−1^.

b BMI, height.

c Cancer, diabetes, cardiovascular disease.

d Own wealth quintile per benefit unit (unit is a couple or single person along with their dependent children), education level.

e Alcohol consumption, smoking, physical activity.
